# Direct probing of single-molecule chemiluminescent reaction dynamics under catalytic conditions in solution

**DOI:** 10.1038/s41467-023-43640-1

**Published:** 2023-12-02

**Authors:** Ziqing Zhang, Jinrun Dong, Yibo Yang, Yuan Zhou, Yuang Chen, Yang Xu, Jiandong Feng

**Affiliations:** 1 Laboratory of Experimental Physical Biology, Department of Chemistry, Zhejiang University, 310058 Hangzhou, China; 2 Research Center for Quantum Sensing, Research Institute of Intelligent Sensing, Zhejiang Lab, 311121 Hangzhou, China

**Keywords:** Imaging studies, Catalysis

## Abstract

Chemical reaction kinetics can be evaluated by probing dynamic changes of chemical substrates or physical phenomena accompanied during the reaction process. Chemiluminescence, a light emitting exoenergetic process, involves random reaction positions and kinetics in solution that are typically characterized by ensemble measurements with nonnegligible average effects. Chemiluminescent reaction dynamics at the single-molecule level remains elusive. Here we report direct imaging of single-molecule chemiluminescent reactions in solution and probing of their reaction dynamics under catalytic conditions. Double-substrate Michaelis–Menten type of catalytic kinetics is found to govern the single-molecule reaction dynamics in solution, and a heterogeneity is found among different catalyst particles and different catalytic sites on a single particle. We further show that single-molecule chemiluminescence imaging can be used to evaluate the thermodynamics of the catalytic system, resolving activation energy at the single-particle level. Our work provides fundamental insights into chemiluminescent reactions and offers an efficient approach for evaluating catalysts.

## Abstract (web summary)

Research into the dynamics of chemical reactions at the single-molecule level is a pivotal undertaking. Here, the authors present a direct investigation of the chemiluminescent reaction dynamics of single molecules in solution, providing spatiotemporally resolved insights into chemical reactions.

## Introduction

Chemical reactions generate products as a result of electron transfer during the forming and breaking of chemical bonds between atoms, which is accompanied by physical phenomena, such as heat conversion and light generation. Observation of chemical reactions can be achieved by monitoring the changes of reactant, the formation of products or probing the accompanied physical effects, which are usually viewed at the ensemble level^1^. Although some studies observing individual chemical reactions have been reported^2,3^, direct probing of chemical reaction positions and their dynamics at the single-molecule level in solution has been a long-standing challenge in chemistry. Due to the faintest physical signals from individual reactions, most of the available single-molecule chemistry approaches measure the property of chemical substance from a reaction output, such as the scanning probe-based approach measuring the conductivity of the substance^4^ and single-molecule fluorescence microscopy monitoring the fluorescence of the transform^5–10^. We recently achieved the direct observation of single-molecule reactions of a specific chemiluminescence phenomenon (electrochemiluminescence) near the electrode surface^11,12^. However, general single-molecule chemiluminescent reaction events initialized by the free diffusion and the stochastic collision of molecules deep in solution can result in large uncertainties of the reaction location and the reaction time. Revealing such chemiluminescent reaction dynamics at the single-molecule level remains challenging.

In this study, we report the direct imaging of single-molecule chemiluminescent reactions in solution and probing of their reaction dynamics under catalytic conditions. Our experiment directly addresses the reaction itself by monitoring the chemiluminescence from the reaction without any background caused by laser and autofluorescence, which is beneficial for probing catalysis.

## Results and discussion

**Fig. 1:**
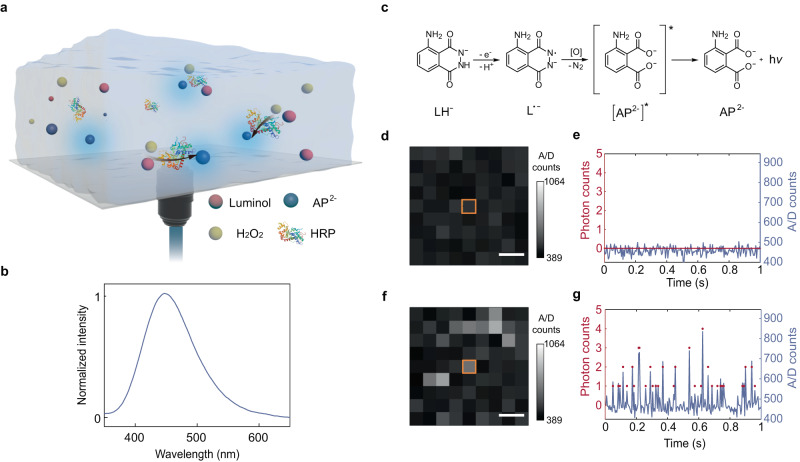
Observation of single-molecule chemiluminescent reactions. **a** Schematic of chemiluminescence imaging system. **b** Chemiluminescence emission spectrum of luminol-H_2_O_2_ system. **c** Mechanism of luminol oxidation and chemiluminescence emission. [O] is the reactive oxygen species for H_2_O_2_ decomposition. **d** Image mode of background. Scale bar: 200 nm. **e** Camera A/D counts and photon counting trace of background. **f** Image mode of single-molecule chemiluminescence signals. Scale bar: 200 nm. **g** A/D counts and photon counting trace of single-molecule chemiluminescence signals. The chemiluminescence signals are observed at 1 mM luminol, 15 mM H_2_O_2,_ 0.23 mM 4-iodophenol, 5 nM HRP with an exposure time of 5 ms and an electron-multiplying gain (EM Gain) of 500. Source data are provided as a Source Data file.

Chemiluminescence is a light emission process, in which a single photon is emitted from a single-molecule chemical reaction, featuring advantages of ultralow background and high sensitivity in imaging^13,14^ and immunoassay^15,16^. A classical chemiluminescence system is used in our experiments (Fig. 1a, b): luminol (5-amino-2,3-dihydro-1,4-phthalazinedione, ${{{{{{\rm{LH}}}}}}}^{-}$) reacts with hydrogen peroxide (H_2_O_2_) under the catalysis of horseradish peroxidase (HRP).

To understand the enzymatic chemiluminescent reactions, we elucidate the mechanism of chemiluminescence based on luminol and H_2_O_2_ with HRP as the catalyst, which is known as^17,18^:

1 $${{{{{\rm{HRP}}}}}}+{{{{{{\rm{H}}}}}}}_{2}{{{{{{\rm{O}}}}}}}_{2}\to {{{{{\rm{HRP}}}}}}-{{{{{\rm{I}}}}}}+{{{{{{\rm{H}}}}}}}_{2}{{{{{\rm{O}}}}}}$$

2 $${{{{{\rm{HRP}}}}}}-{{{{{\rm{I}}}}}}+{{{{{{\rm{LH}}}}}}}^{-}\to {{{{{\rm{HRP}}}}}}-{{{{{\rm{II}}}}}}+{{{{{{\rm{L}}}}}}}^{{{\cdot }}-}$$

3 $${{{{{\rm{HRP}}}}}}-{{{{{\rm{II}}}}}}+{{{{{{\rm{LH}}}}}}}^{-}\to {{{{{\rm{HRP}}}}}}+{{{{{{\rm{L}}}}}}}^{{{\cdot }}-}$$

4 $${{{{{{\rm{L}}}}}}}^{{{\cdot }}-}\to {{{{{{\rm{AP}}}}}}}^{2-}+{{{{{\rm{h}}}}}}\upsilon$$

HRP is first oxidized to HRP-I (one of the peroxidase reactive intermediates) by H_2_O_2_, while this product reacts with two molecules of luminol anions sequentially to produce two luminol ionic radicals (${{{{{{\rm{L}}}}}}}^{{{\cdot }}-}$), which are further oxidized to the excited state of 3-aminophthalic acid (${[{{{{{{\rm{AP}}}}}}}^{2-}]}^{*}$) leading to chemiluminescence^19^, as shown in Fig. 1c. A detailed structure and explanation of these abbreviations is shown in Supplementary Table 1. 4-iodophenol can be introduced as an enhancer to increase the chemiluminescence intensity. 4-iodophenol’s phenoxyl radicals can oxidize luminol and act as a redox mediator role; on the other hand, 4-iodophenol can react rapidly with the peroxidase reactive intermediates (HRP-I and HRP-II), thus accelerating the enzyme turnover frequency^17,20,21^. To image single-molecule chemiluminescence reactions and study the chemiluminescence kinetics, we added free HRP to the reaction system which is consistent with solution environments in the bulk assays^14,15,17^, and elevated the focal plane into solution (rather than the surface of the glass coverslip) for detection. The emitted photons are then collected through the optical lens and detected by an electron-multiplying charge-coupled device (EMCCD) camera that enables high signal-noise ratio (SNR) single-photon detection.

To visualize a single event, we have employed a single-molecule spatiotemporal isolation strategy that controls the molecular density as well as the acquisition conditions for separating individual reactions in both space and time^1^. As chemiluminescence does not require laser excitation, the system background can be minimized to the detector offset level (Fig. 1d, e), which greatly facilitates the single-molecule observation with a high SNR (≈5). Figure 1f, g illustrates a typical on-off turnover chemiluminescence trajectory from a single pixel, a classic signature of single-molecule signals from stochastic reactions. The discrete number of acquired photons (1, 2, 3, …) converted from the camera analog to digital (A/D) counts further indicates the observation of single-molecule reactions as a single photon is the output of an individual reaction. Thus, we managed to directly probe general single-molecule chemiluminescent reactions in solution, and our single-molecule chemiluminescence method can provide advantages with high spatiotemporal resolution compared with the bulk assays^14,15^.

### Single-molecule analysis of HRP-catalyzed reactions

**Fig. 2:**
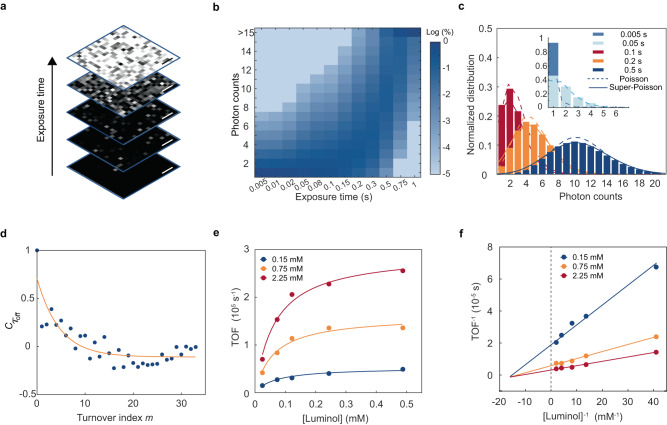
Single-molecule analysis of HRP-catalyzed chemiluminescent reactions. **a** Images at increasing exposure times. Scale bar: 400 nm. **b** Statistics of photon counts per pixel for different exposure times. **c** Poisson and super-Poisson distribution of photon counts. Lambda is defined here as the variance and expectation of the distribution. The lambda value is 0.09, 0.95, 1.71, 4.12, 9.84 under the exposure time of 0.005 s, 0.05 s, 0.1 s, 0.2 s, 0.5 s, respectively. **d** Autocorrelation function C_τ_ of τ_off_ from a single-molecule turnover trajectory. The *x* axis is the turnover index. The yellow solid line is the fit with a single exponential for decay constant m_off_ of 0.214 detected turnovers. **e** TOF as a function of luminol concentration and the Michaelis-Menten equation fitting under three H_2_O_2_ concentrations. **f** Single-molecule Lineweaver-Burk plot corresponding to (**e**). The three lines intersect at a point to the left of the dashed line (abscissa <0). The chemiluminescence signal for different exposure times is acquired under 2.5 mM luminol, 15 mM H_2_O_2_, 0.23 mM 4-iodophenol, 5 nM HRP with EM Gain of 500. The chemiluminescence signal for different concentrations is acquired under 0.23 mM 4-iodophenol, 2.5 nM HRP with an exposure time of 5 ms and an EM Gain of 500. Source data are provided as a Source Data file.

To understand the statistical nature of single-molecule reactions, we performed imaging under different exposure times and analyzed the obtained photon number distribution in single pixels (Fig. 2a, b). The interval time between two events is defined as τ_off_, which is the time for product formation and contains abundant kinetic information of the chemiluminescent reactions. With the increase of exposure time, the number of accumulated photons increases (Supplementary Fig. 1, 2). As shown in Fig. 2c and Supplementary Fig. 3, photon number distribution shows a stochastic nature of chemiluminescent reactions at different exposure times, which initially follows a Poisson distribution and then transits to a super-Poisson distribution, indicating the reaction activity fluctuations.

The activity fluctuations are usually reflected by the reaction rate of the product formation process. We extracted τ_off_ from the single-molecule turnover trajectories and calculated the autocorrelation function^22^, which displays an evident exponential decay with a decay constant of 0.214 detected turnovers, and further suggests the existence of activity fluctuations^22^ (Fig. 2d).

From the stochastic single-molecule reaction trajectory, kinetic information of reactions can be obtained from the τ_off_ between two adjacent events (Supplementary Fig. 4, 5) or the turnover frequency (TOF), the number of photon counts per unit time^23^,

5 $${{{{{\rm{TOF}}}}}}=\frac{{{{{{{\rm{\varphi }}}}}}}_{{{{{{\rm{r}}}}}}}{{{{{{\rm{\varphi }}}}}}}_{{{{{{\rm{q}}}}}}}{{{{{{\rm{\varphi }}}}}}}_{{{{{{\rm{c}}}}}}}{{{{{{\rm{\varphi }}}}}}}_{{{{{{\rm{d}}}}}}}{N}_{{{{{{\rm{m}}}}}}}}{t}=\frac{{N}_{{{{{{\rm{d}}}}}}}}{t}$$

where ${{{{{{\rm{\varphi }}}}}}}_{{{{{{\rm{r}}}}}}}$ is the efficiency of the reaction, ${{{{{{\rm{\varphi }}}}}}}_{{{{{{\rm{q}}}}}}}$ is the luminescence quantum yield (determined by the luminescent substrate), ${{{{{{\rm{\varphi }}}}}}}_{{{{{{\rm{c}}}}}}}$ is the photon collection efficiency, ${{{{{{\rm{\varphi }}}}}}}_{{{{{{\rm{d}}}}}}}$ is the photoelectric conversion efficiency of EMCCD, ${N}_{{{{{{\rm{m}}}}}}}$ is the number of molecules in collision, ${N}_{{{{{{\rm{d}}}}}}}$ is the total number of detected photons, and *t* is the acquisition time. The total efficiency in chemiluminescence is limited as all above efficiencies need to be considered. Based on τ_off_ and TOF analysis, we could explore the dynamics of single-molecule chemiluminescent reaction. In the presence of enhancer 4-iodophenol, we fixed the concentration of hydrogen peroxide (at 0.15, 0.75, and 2.25 mM) to figure out the luminol concentration dependence of the reaction kinetics. As shown in Fig. 2e, the single-molecule reaction kinetics evaluated under the HRP catalysis suggests an increasing TOF that tends to be stable with the increase of luminol concentration. Similarly, with a fixed concentration of luminol, the corresponding TOF also increases gradually with the growing H_2_O_2_ concentration. The obtained Lineweaver-Burk fitting result at the single-molecule level conforms to the characteristics of the double-substrate ‘sequential’ mechanism of Michaelis-Menten equation^24,25^ (Fig. 2f),

6 $${{{{{\rm{TOF}}}}}}=\frac{{V}_{\max }[{{{{{\rm{H}}}}}}][{{{{{\rm{L}}}}}}]}{{K}_{{{{{{\rm{m}}}}}}}^{{{{{{\rm{H}}}}}}}[{{{{{\rm{L}}}}}}]+{K}_{{{{{{\rm{m}}}}}}}^{{{{{{\rm{L}}}}}}}[{{{{{\rm{H}}}}}}]+{K}_{{{{{{\rm{iH}}}}}}}{K}_{{{{{{\rm{m}}}}}}}^{{{{{{\rm{L}}}}}}}+[{{{{{\rm{H}}}}}}][{{{{{\rm{L}}}}}}]}$$

where the ${V}_{\max }$ is the maximum reaction rate, ${K}_{{{{{{\rm{m}}}}}}}$ is the Michaelis-Menten constant, $[{{{{{\rm{H}}}}}}]$ and $[{{{{{\rm{L}}}}}}]$ are the concentration of H_2_O_2_ and luminol, ${K}_{{{{{{\rm{iH}}}}}}}$ is the dissociation constant of H_2_O_2_ and the enzyme HRP. The ‘sequential’ reaction mechanism scheme of double-substrate Michaelis-Menten equation is discussed in Supplementary Fig. 6. With this model, ${K}_{{{{{{\rm{m}}}}}}}$ of luminol at different H_2_O_2_ concentrations was obtained (Supplementary Table 2). Similar results of the reaction kinetics were obtained from τ_off_ analysis (Supplementary Fig. 7) and power spectral density (PSD) analysis (Supplementary Fig. 8).

To further probe the reaction itself, we removed the enhancer. The ${{{{{\rm{TOF}}}}}}$ also increases with increasing concentration of luminol and H_2_O_2_, similar to the case with enhancer (Supplementary Fig. 9). However, the Lineweaver-Burk fitting shows a parallel relationship, suggesting a ‘ping-pong’ mechanism for the reaction of luminol and H_2_O_2_^24,26^ (Supplementary Table 3). Moreover, chemiluminescence intensity without enhancer is 1 or 2 orders of magnitude lower than that with the presence of enhancer. Based on the experimental results and the role of 4-iodophenol for oxidating luminol^17^, we propose a possible route leading to the mechanism shift (Supplementary Fig. 10).

### Single-molecule reaction kinetics on hematin particles

**Fig. 3:**
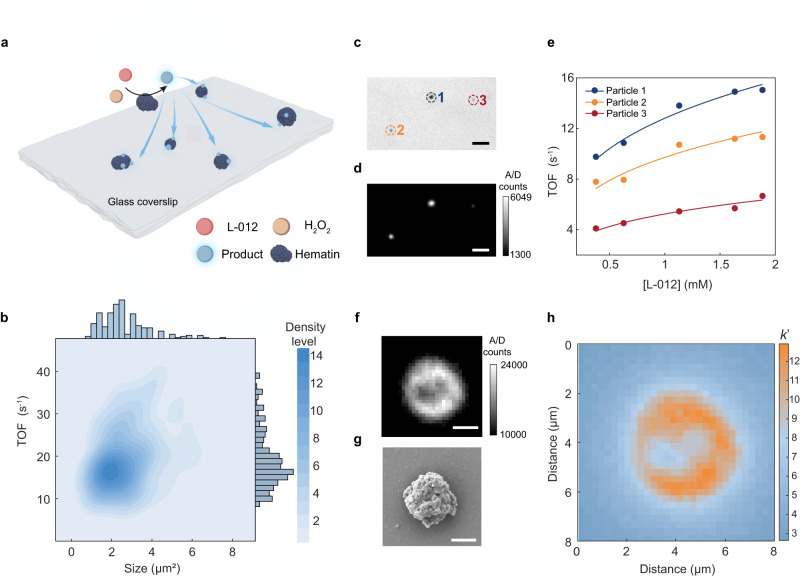
Kinetics of hematin-catalyzed single-molecule chemiluminescent reactions. **a** Schematic of hematin-catalyzed chemiluminescence imaging system. **b** Statistics of catalytic photon counts for over 200 hematin particles. **c** Bright field image and (**d**) chemiluminescence image of three different particles. Scale bar: 6 μm. **e** TOF as a function of L-012 concentration and kinetic curves fitting for three different hematin particles of (**c**). **f** Chemiluminescence image and (**g**) SEM image of a single hematin particle. Scale bar: 2 μm. **h** Transformed reaction rate constant (*k*’) mapping per pixel of corresponding sites of (**f**). Source data are provided as a Source Data file.

Using this approach, we are able to observe single-molecule chemiluminescent reactions in solution with freely diffusing HRP molecules or on single HRP particles (Supplementary Fig. 11). However, we did not manage to observe single-molecule chemiluminescent reaction emission on single HRP molecules immobilized on a glass coverslip which is limited by the efficiency of the current chemiluminescence system (see results and discussions in Supplementary Fig. 12). The structure of HRP is often affected by the environment of chemiluminescence such as pH, ionic strength and temperature^27^, which limits its catalytic activity and operations. Hematin, as the active center of HRP, features good stability and activity for catalyzing chemiluminescent reactions^28,29^. We immobilized hematin porcine catalyst particles on glass and performed chemiluminescence imaging for kinetics analysis. L-012 (8-amino-5-chloro-7-phenylpyrido[3,4-d]pyridazine-1,4(2H,3H)dione), which is the analogue of luminol with higher luminescence efficiency, is employed here as the luminescence substrate. L-012 reacts with H_2_O_2_ under the catalysis of hematin and generates light at 480 nm in this process (Fig. 3a and Supplementary Fig. 13). The chemiluminescence generated on hematin particles features a good stability, facilitating control experiments and correlative imaging (Supplementary Fig. 14). Compared with bright field, scanning electron microscopy (SEM), atomic force microscopy (AFM) imaging and energy dispersive spectrometer (EDS) mapping, an obvious diffusion can be seen in chemiluminescence images (Supplementary Figs. 15, 16), which is attributed to the long lifetime of the excited state of luminol and H_2_O_2_ intermediate radical^30^. According to the distribution of chemiluminescence, diffusion distance can be determined from imaging, and then lifetime of corresponding radicals (12.5 ms for H_2_O_2_ intermediate’s radical and 18.9 ms for L-012 intermediate’s radical) can be obtained (Supplementary Fig. 17). The SEM image shows that the catalyst particles are agglomerated by many strips of fine crystals, forming different shapes and sizes. On single catalyst particles, the distribution of photon number with the change of exposure time also conforms to a Poisson distribution (Supplementary Fig. 18). Statistical analysis of the size and TOF of all imaged hematin particles are shown in Fig. 3b. Based on the spatially resolved imaging, we can target hematin particles of interest for analysis of reaction kinetics (Fig. 3c–e):

7 $$\,{{{{{\rm{TOF}}}}}}={{{{{\rm{\varphi }}}}}}k{\left[{{{{{\rm{L}}}}}}\right]}^{{{{{{\rm{m}}}}}}}{[{{{{{\rm{H}}}}}}]}^{{{{{{\rm{n}}}}}}}={k}^{{\prime} }{\left[{{{{{\rm{L}}}}}}\right]}^{{{{{{\rm{m}}}}}}}$$

where ${{{{{\rm{\varphi }}}}}}$ is the total efficiency, *k* is the reaction rate constant, $[{{{{{\rm{H}}}}}}]$ and $[{{{{{\rm{L}}}}}}]$ are the concentration of L-012 and H_2_O_2_, m/n is the index item, and ${k}^{{\prime} }$ is the transformed reaction rate constant. When the H_2_O_2_ concentration is fixed, the kinetics on hematin follows the Eq. (7), and the spatially resolved kinetics can be used to evaluate the heterogeneity across different catalyst particles.

Moreover, we selected a single catalyst particle for obtaining their chemiluminescence kinetics at different concentrations of L-012 (Fig. 3f–h, Supplementary Fig. 19). As shown in Fig. 3f, g, the single particle shows different catalytic behavior at different sites. Single-molecule kinetics at sub-particle sites follows the kinetics of Eq. (7) with the increasing L-012 concentration, but reveals different chemiluminescent reaction rate constant ${k}^{{\prime} }$ on diverse sites, which reflects the catalytic heterogeneity at the sub-particle level (Fig. 3h).

### Single-molecule reaction thermodynamics on hematin particles

**Fig. 4:**
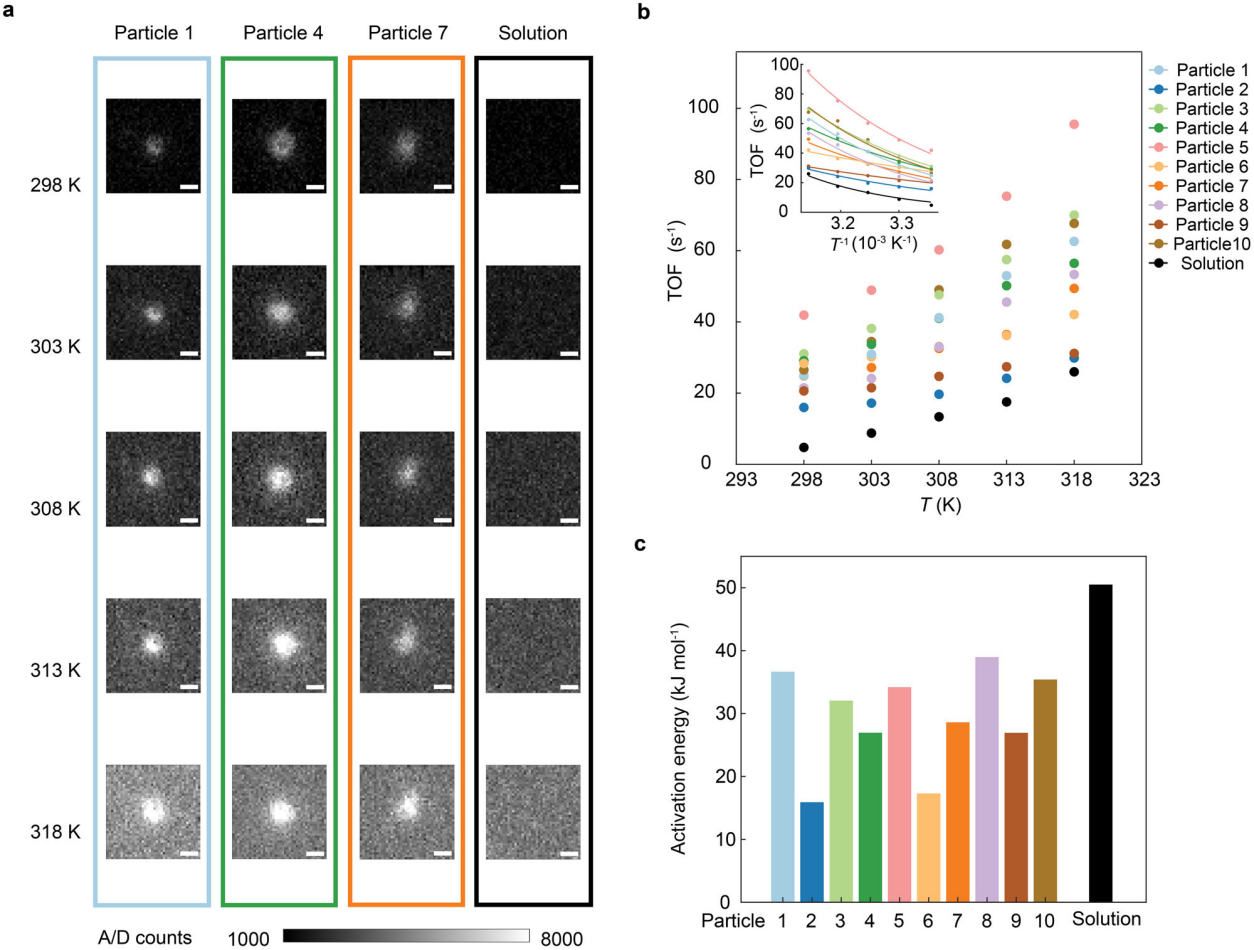
Thermodynamics of hematin-catalyzed single-molecule chemiluminescent reactions. **a** Chemiluminescence images of selected three hematin particles from (**b**) and solution at five different temperatures. Scale bar: 2 μm. **b** TOF on ten particles and in solution at different temperatures and corresponding Arrhenius equation fitting (the insert figure). **c** Activation energy extracted from (**b**). Source data are provided as a Source Data file.

The ability to reduce the activation energy of the reaction system is a crucial indicator in evaluating the catalyst. We performed temperature-dependent experiments of the chemiluminescence reaction with hematin to explore the thermodynamics of this reaction (Supplementary Fig. 20). Figure 4a shows the chemiluminescence images under different temperatures of the solution and three representative catalyst particles (selected from ten particles in Fig. 4b). The thermodynamics of ten particles is obtained in Fig. 4b. As the temperature increases, TOF within the catalyst-free solution area increases rapidly with temperature while TOF of ten particles increases with a slower trend. Based on the TOF-temperature relationship, the activation energy can be estimated from the Arrhenius relationship (the insert figure in Fig. 4b)^31^,

8 $$\,{{{{{\rm{TOF}}}}}}={A}^{{\prime} }{{{{{{\rm{e}}}}}}}^{\frac{-{E}_{{{{{{\rm{a}}}}}}}}{{{{{{\rm{R}}}}}}T}}$$

where$\,{E}_{{{{{{\rm{a}}}}}}}$ is the activation energy, *T* is the temperature, R is the molar gas constant, and ${A}^{{\prime} }$ is the transformed coefficient (see Methods). The calculated activation energy of the reaction in solution is 50.5 kJ mol^−1^, and of these ten hematin particles is in the range of 10–40 kJ mol^−1^ (Fig. 4c), which is in agreement with the activation energy range reported in the literature^32^.

Our approach probes single-molecule chemiluminescent reaction kinetics in solution with freely diffusing HRP or on single catalyst particles with a single-photon sensitivity down to single-molecule reaction level, and spatially evaluates the thermodynamics of different particles, which can be used for investigating the relationships between the structures and the catalytic activity of catalysts. This method directly probes the light output of the chemical reaction, which minimizes the measurement background and the interference to the reaction system. The information obtained by the single-molecule chemiluminescence approach provides fundamental insights into the heterogeneity of both the kinetics and thermodynamics of catalytic reactions on single-particle level, offering a single-molecule methodology for studying chemical processes.

## Methods

### Single-molecule chemiluminescence measurement setup

Single-molecule chemiluminescence measurements were performed on an inverted optical microscope (IX83, Olympus). Chemiluminescence generated by the reactants was collected by a 150× oil objective (Olympus, numerical aperture: 1.45) for HRP-catalyzed experiments or a 60× oil objective (Olympus, numerical aperture: 1.49) for hematin porcine-catalyzed experiments, respectively, and detected by an EMCCD camera (iXon Ultra 897, Andor) with an EM Gain of 500.

### Detection of single-molecule chemiluminescence in solution

The glass coverslip used for holding aqueous sample solution was sonicated in acetone, isopropanol, and deionized water sequentially for 30 min, followed by nitrogen drying, and then fixed into the microscope sample chamber. 2 mL-volume mixture of Tris-HCl buffer (0.1 M, pH = 8.5), HRP (ThermoFisher Scientific), luminol (C_8_H_7_N_3_O_2_, Aladdin, 98%) and 4-iodophenol (C_6_H_5_IO, Macklin, 98%) solution was added to the sample chamber, and H_2_O_2_ solution was added to the whole solution which was then prepared under stirring at room temperature for further experiments.

### Chemiluminescence imaging of hematin porcine particles

First, hematin porcine (C_34_H_33_N_4_O_5_Fe, Sigma, 96%) powder was dispersed in ethanol and treated with a 20-min-long ultrasound process for homogenizing, then 100 μL hematin solution was added onto the glass coverslip and kept for 20 min until the ethanol evaporated completely, and the unbound particles could be rinsed with deionized water. The glass coverslip was then fixed into the PTFE sample chamber with a gasket, and 2 mL mixture of Tris-HCl buffer and L-012 solution (C_13_H_9_ClN_4_O_2_, Fujifilm, 90%) was added, followed by adding H_2_O_2_ solution and fully stirring to start the reaction. Detection was performed after the signal stabilizes for 1–2 min.

### Temperature-changing experiment for hematin porcine particles

Hematin porcine particles were deposited on glass coverslip. The polyimide heating film was wrapped on the outer surface of the sample chamber, and was connected to the temperature control system. After adding buffer and substrate solution, the temperature of the solution was heated to the set value via the controller, afterwards the chemiluminescence was detected by an EMCCD.

### Correlation of characterization methods

To carry out the correlation experiments, an array of Cr-Au was fabricated on the glass coverslip for navigation. Then the bright field and chemiluminescence images were taken. In addition, SEM (ZEISS Gemini 300) and AFM (Molecular Vista VistaScope) images were acquired to obtain the morphology and structure of catalyst particles. EDS mapping was used to determine the composition of the catalyst.

### Autocorrelation analysis

We analyzed the autocorrelation using following equation,

9 $${C}_{\tau }\left({{{{{\rm{m}}}}}}\right)= < \Delta \tau \left(0\right)\Delta \tau \left({{{{{\rm{m}}}}}}\right) > / < \Delta {\tau }^{2} > $$

where m is the turnover index of the sequence, and ${C}_{\tau }\left({{{{{\rm{m}}}}}}\right)$ is the autocorrelation function.

### Transformation of the Arrhenius equation

10 $${{{{{\rm{TOF}}}}}}={{{{{\rm{\varphi }}}}}}{\left[{{{{{\rm{L}}}}}}\right]}^{{{{{{\rm{m}}}}}}}{[{{{{{\rm{H}}}}}}]}^{{{{{{\rm{n}}}}}}}\times k={{{{{\rm{\varphi }}}}}}{\left[{{{{{\rm{L}}}}}}\right]}^{{{{{{\rm{m}}}}}}}{[{{{{{\rm{H}}}}}}]}^{{{{{{\rm{n}}}}}}}\times A{{{{{{\rm{e}}}}}}}^{\frac{-{E}_{{{{{{\rm{a}}}}}}}}{{{{{{\rm{R}}}}}}T}}={A}^{{\prime} }{{{{{{\rm{e}}}}}}}^{\frac{-{E}_{{{{{{\rm{a}}}}}}}}{{{{{{\rm{R}}}}}}T}}$$

with *E*_a_: activation energy, *A*: pre-exponential factor, *T*: temperature, R: molar gas constant, ${A}^{{\prime} }$: the transformed coefficient.

### Reporting summary

Further information on research design is available in the Nature Portfolio Reporting Summary linked to this article.

### Supplementary information

Supplementary Information

Peer Review File

Reporting Summary

### Source data

Source Data

## Data Availability

The data that support the findings of this study are available from the corresponding authors upon request. Source data are provided with this paper.

## References

[1] Feng J (2022). Electrochemistry probed one molecule at a time. Curr. Opin. Electrochem..

[2] Collinson MM (1995). Observation of individual chemical reactions in solution. Science.

[3] Li, Y. et al. Visualizing the intermittent gating of Na^+^/H^+^ antiporters in single native bioluminescent bacteria. *Angew. Chem. Int. Ed*. **62**, e202215800 (2022). 10.1002/anie.202215800 36562656

[4] de Oteyza DG (2013). Direct imaging of covalent bond structure in single-molecule chemical reactions. Science.

[5] Mao X (2022). Inter-facet junction effects on particulate photoelectrodes. Nat. Mater..

[6] Liu X (2022). Revealing the catalytic kinetics and dynamics of individual Pt atoms at the single-molecule level. Proc. Natl. Acad. Sci..

[7] Xiao Y (2023). Single-molecule fluorescence imaging for probing nanocatalytic process. Chem.

[8] Whiting GT (2019). Visualizing pore architecture and molecular transport boundaries in catalyst bodies with fluorescent nanoprobes. Nat. Chem..

[9] Fu D (2022). Unravelling channel structure–diffusivity relationships in zeolite zsm-5 at the single-molecule level. Angew. Chem. Int. Ed..

[10] Kumar N (2019). Nanoscale chemical imaging of a single catalyst particle with tip-enhanced fluorescence microscopy. ChemCatChem.

[11] Dong J (2021). Direct imaging of single-molecule electrochemical reactions in solution. Nature.

[12] Dong J (2022). Operando imaging of chemical activity on gold plates with single-molecule electrochemiluminescence microscopy. Angew. Chem. Int. Ed..

[13] Lee D (2007). In vivo imaging of hydrogen peroxide with chemiluminescent nanoparticles. Nat. Mater..

[14] Zheng T (2022). A chemical timer approach to delayed chemiluminescence. Proc. Natl. Acad. Sci..

[15] Whitehead TP (1983). Enhanced luminescence procedure for sensitive determination of peroxidase-labelled conjugates in immunoassay. Nature.

[16] Yuan H (2012). Chemical molecule-induced light-activated system for anticancer and antifungal activities. J. Am. Chem. Soc..

[17] Easton PM (1996). Quantitative model of the enhancement of peroxidase-induced luminol luminescence. J. Am. Chem. Soc..

[18] Lind J (1983). Chemiluminescence mechanism of cyclic hydrazides such as luminol in aqueous solutions. J. Am. Chem. Soc..

[19] Nakamura M (1998). One- and two-electron oxidations of luminol by peroxidase systems. Free Radic. Biol. Med..

[20] Lee J (1965). Absolute spectral sensitivity of phototubes and the application to the measurement of the absolute quantum yields of chemiluminescence and bioluminescence. Photochem. Photobiol..

[21] Liu Y (2017). Firefly-mimicking intensive and long-lasting chemiluminescence hydrogels. Nat. Commun..

[22] Xu W (2008). Single-molecule nanocatalysis reveals heterogeneous reaction pathways and catalytic dynamics. Nat. Mater..

[23] Kozuch S (2012). “Turning over” definitions in catalytic cycles. ACS Catal..

[24] Cook, P. F. & Cleland, W. W. *Enzyme Kinetics and Mechanism* (Garland Science, 2007).

[25] Cormier MJ (1968). An investigation of the mechanism of the luminescent peroxidation of luminol by stopped flow techniques. J. Biol. Chem..

[26] Cleland WW (1963). The kinetics of enzyme-catalyzed reactions with two or more substrates or products: III. Prediction of initial velocity and inhibition patterns by inspection. Biochim. Biophys. Acta Spec. Sect. Enzymol. Subj..

[27] Gao L (2007). Intrinsic peroxidase-like activity of ferromagnetic nanoparticles. Nat. Nanotechnol..

[28] Neufeld HA (1965). Chemiluminescence of luminol in the presence of hematin compounds. Anal. Biochem..

[29] Liu Y (1995). Chemiluminescence and epr studies on the excitation site of ferric-heme-oxo complexes of natural and model heme systems. Photochem. Photobiol..

[30] Roda A (1998). Chemiluminescent imaging of enzyme-labeled probes using an optical microscope–videocamera luminograph. Anal. Biochem..

[31] Arrhenius, S. Über die Reaktionsgeschwindigkeit bei der Inversion von Rohrzucker durch Säuren. *Z. Phys. Chem.* **4**, 226–248 (1889).

[32] Craig DB (1996). Studies on single alkaline phosphatase molecules: reaction rate and activation energy of a reaction catalyzed by a single molecule and the effect of thermal denaturationthe death of an enzyme. J. Am. Chem. Soc..

