# Publisher Correction: Direct probing of single-molecule chemiluminescent reaction dynamics under catalytic conditions in solution

**DOI:** 10.1038/s41467-024-45496-5

**Published:** 2024-02-05

**Authors:** Ziqing Zhang, Jinrun Dong, Yibo Yang, Yuan Zhou, Yuang Chen, Yang Xu, Jiandong Feng

**Affiliations:** 1 Laboratory of Experimental Physical Biology, Department of Chemistry, Zhejiang University, 310058 Hangzhou, China; 2 Research Center for Quantum Sensing, Research Institute of Intelligent Sensing, Zhejiang Lab, 311121 Hangzhou, China

**Keywords:** Imaging studies, Catalysis

Correction to: *Nature Communications* 10.1038/s41467-023-43640-1, published online 02 December 2023

The original PDF version of this article contained an error in Fig. 1, where panel e inadvertently displayed a red dot that was not pertinent to the data. The HTML version was unaffected. The original article has been corrected.

