# Supplementary material for: Direct probing of single-molecule chemiluminescent reaction dynamics under catalytic conditions in solution
Source: Nat Commun. 2023 Dec 2;14:7993. doi: 10.1038/s41467-023-43640-1 (PMC10693624; doi:10.1038/s41467-023-43640-1)
Supplement: Supplementary file 1 — Supplementary Information [file 41467_2023_43640_MOESM1_ESM.pdf]

Supplementary Information for
Direct probing of single-molecule chemiluminescent reaction dynamics under catalytic conditions in solution

Ziqing Zhang[1], Jinrun Dong[1], Yibo Yang[1], Yuan Zhou[1], Yuang Chen[1], Yang Xu[1],

Jiandong Feng[1,2]*

[1]Laboratory of Experimental Physical Biology, Department of Chemistry, Zhejiang University, 310058, Hangzhou, China

[2]Research Center for Quantum Sensing, Research Institute of Intelligent Sensing, Zhejiang Lab, 311121, Hangzhou, China

**Correspondence should be addressed to J.F. jiandong.feng@zju.edu.cn*

Table of contents

Supplementary Information 1

Supplementary Figures 4

Suppl. Fig. 1. Time trajectory of a random selected single pixel at different exposure times. 4

Suppl. Fig. 2. Signal saturation for different exposure times. 5

Suppl. Fig. 3. Photon count distribution at different time windows. 6

Suppl. Fig. 4. Distribution of τ_{off} in a single pixel at different luminol concentrations. 7

Suppl. Fig. 5. Distribution of τ_{off} over all time trajectories of pixels in the field of view. 8

Suppl. Fig. 6. 'Sequential' reaction mechanism scheme of double-substrate Michaelis-Menten equation. 9

Suppl. Fig. 7. Reaction kinetics characterized with τ_{off}. 10

Suppl. Fig. 8. PSD analysis of single-molecule chemiluminescence signals at different luminol concentrations. 11

Suppl. Fig. 9. 'Ping-pong' double-substrate enzyme mechanism and the Lineweaver-Burk plot. 12

Suppl. Fig. 10. 'Ping-pong' and 'sequential' reaction mechanism scheme in this work.. 14

Suppl. Fig. 11. Catalytic behavior on single HRP particles. 16

Suppl. Fig. 12. Immobilized HRP molecule detection in chemiluminescence and

fluorescence on glass coverslip..18

Suppl. Fig. 13. Chemiluminescence emission spectrum of L-012.. 20

Suppl. Fig. 14. Stability of chemiluminescence intensity on hematin particles. .21

Suppl. Fig. 15. Correlated characterizations of a hematin particle......................22

Suppl. Fig. 16. Correlated characterizations of four hematin particles.. 23

Suppl. Fig. 17. Chemiluminescence intensity distribution and diffusion analysis on the hematin particle...24

Suppl. Fig. 18. Photon count distribution on a single hematin particle...............26

Suppl. Fig. 19. Chemiluminescence intensity distribution for the single particle in Fig. 3e. 27

Suppl. Fig. 20. Chemiluminescence stability on the hematin particle…28

Suppl. Table 1. Abbreviations and corresponding explanations.........................29

Suppl. Table 2. Calculated parameters of 'Sequential' mechanism in Fig. 2f. 30

Suppl. Table 3. Calculated parameters of 'ping-pong' mechanism in Suppl. Fig. 9. 31

Supplementary References...32

Supplementary Figures

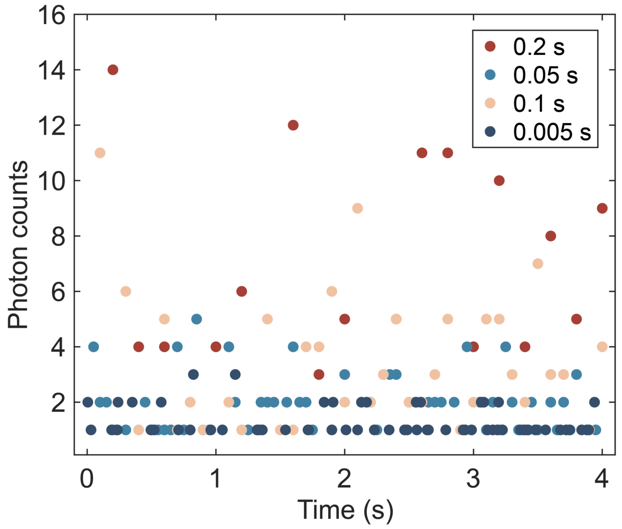

Suppl. Fig. 1. Time trajectory of a random selected single pixel at different exposure times. Concentration conditions: 2.5 mM luminol, 15 mM H_2O_2, 0.23 mM 4-iodophenol, 5 nM HRP, EM Gain: 500. Exposure time: 0.005s, 0.05s, 0.1s, 0.2s. Source data are provided as a Source Data file.

With the increase of exposure time, the number of photons increases gradually with the transition from individual photons (0 and 1) to saturation.

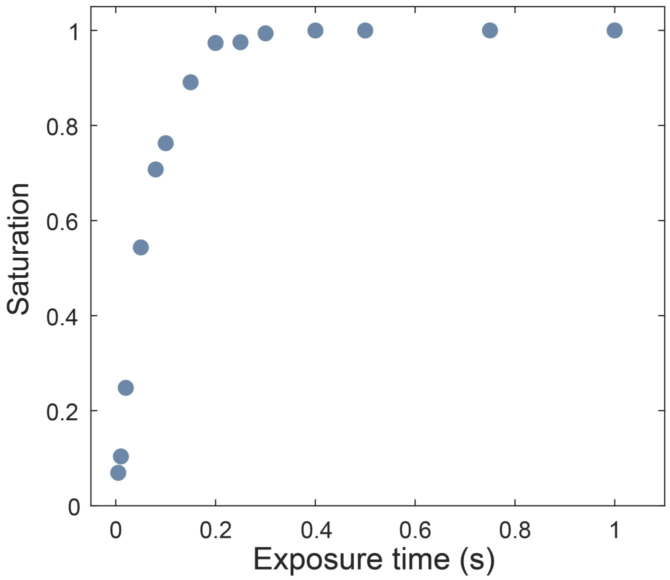

Suppl. Fig. 2. Signal saturation for different exposure times. Source data are provided as a Source Data file.

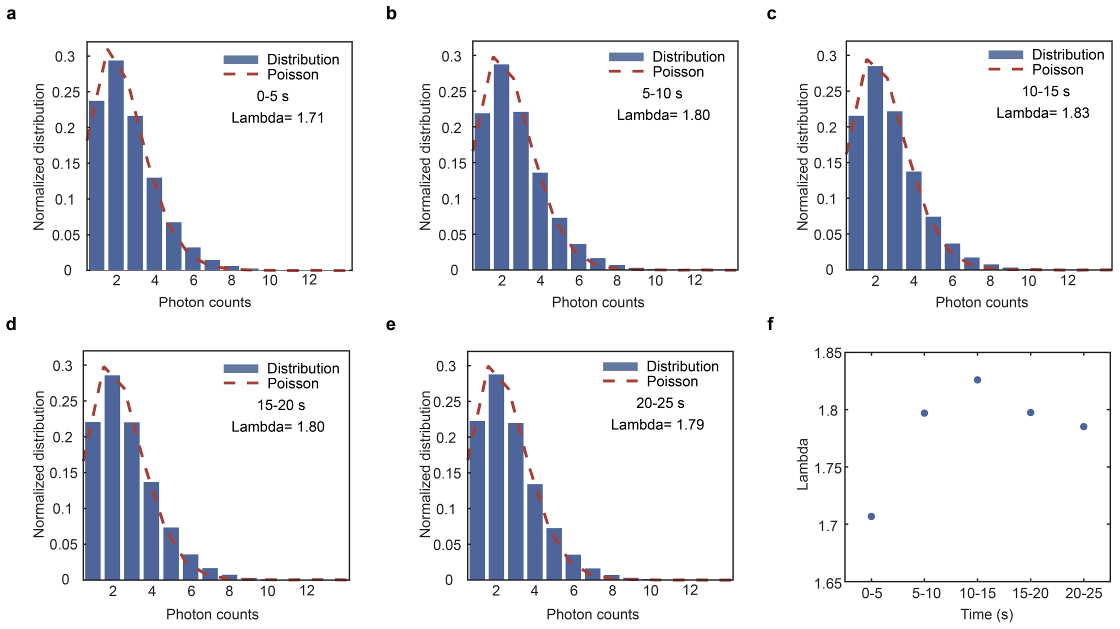

Suppl. Fig. 3. Photon count distribution at different time windows. (a-e) Distribution of photon number at period of 0-5 s, 5-10 s, 10-15 s, 15-20 s, 20-25 s. **(f)** Lambda of distribution during the five periods. Exposure time= 0.1 s. Concentration: 2.5 mM luminol, 15 mM H_2O_2, 0.23 mM 4-iodophenol, 5 nM HRP, EM Gain: 500. Source data are provided as a Source Data file.

Over time, the number of photons follows a Poisson distribution with slight fluctuations. The lambda is 1.71, 1.80, 1.83, 1.80, 1.79 in 0-5 s, 5-10 s, 10-15 s, 15-20 s, 20-25 s, respectively.

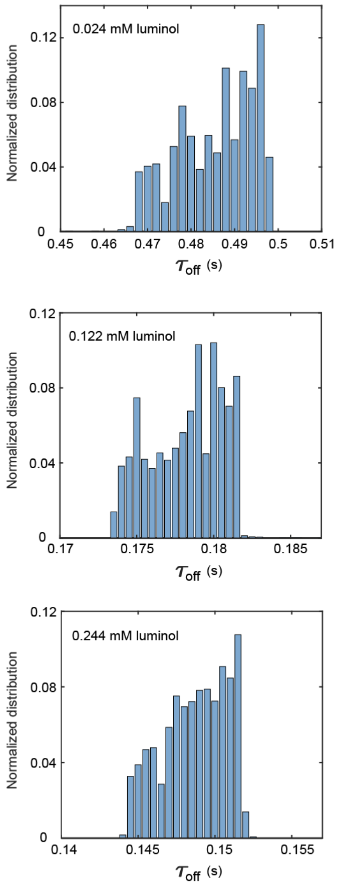

Suppl. Fig. 4. Distribution of τ_{off} in a single pixel at different luminol concentrations. Concentration of luminol: 0.024 mM, 0.122 mM, 0.244 mM. Source data are provided as a Source Data file.

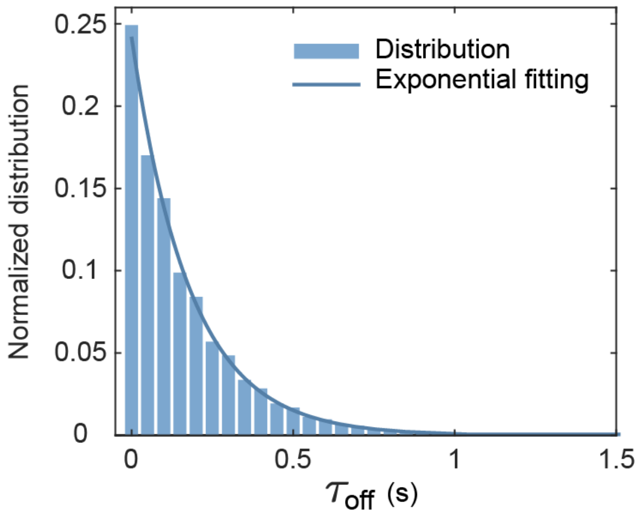

Suppl. Fig. 5. Distribution of τ_{off} over all time trajectories of pixels in the field of view. Source data are provided as a Source Data file.

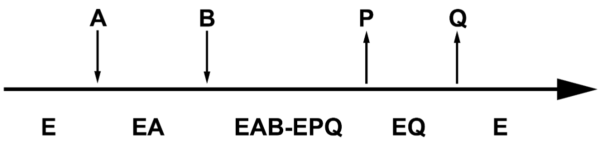

Suppl. Fig. 6. 'Sequential' reaction mechanism scheme of double-substrate Michaelis-Menten equation. E: enzyme, A: first substrate, B: second substrate, P: product from A, Q: product from B. Referring to Cleland, W.W. et al[1], first substrate A combines with enzyme E to form EA, EA combines with the second substrate B to form ternary complex EAB, which can only come from EA, but not EB. The functional groups of A and B are transferred to E to form EPQ, which then releases P and Q to form E. Lineweaver-Burk plot shows that three lines intersect at one point, which is the typical characteristics of the 'sequential' reaction mechanism.

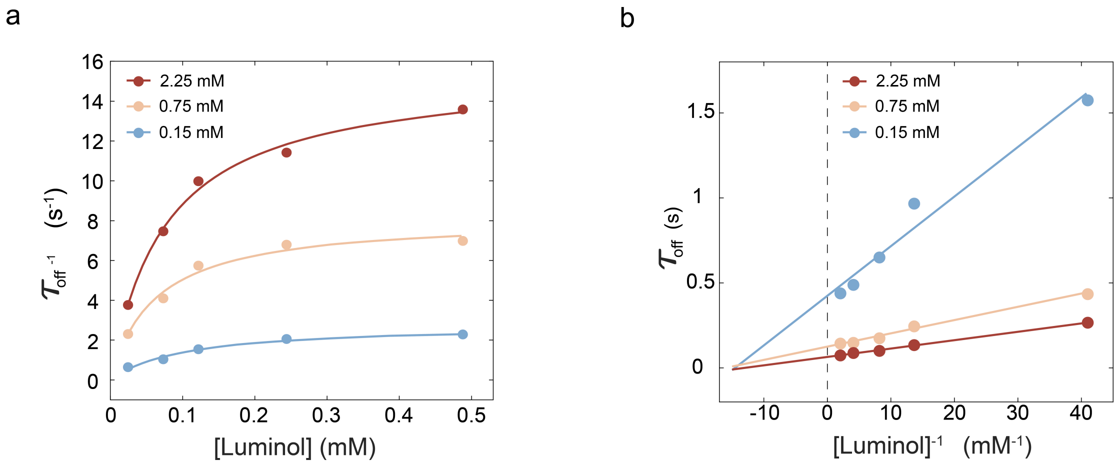

Suppl. Fig. 7. Reaction kinetics characterized with τ_{off}. (a) Chemiluminescence reaction rates and Michaelis-Menten equation fitting of 'sequential' reaction mechanism at different luminol and H_2O_2 concentrations. **(b)** Single-molecule Lineweaver-Burk plot with τ_{off}. The three lines intersect at a point to the left of the dashed line (abscissa < 0). Source data are provided as a Source Data file.

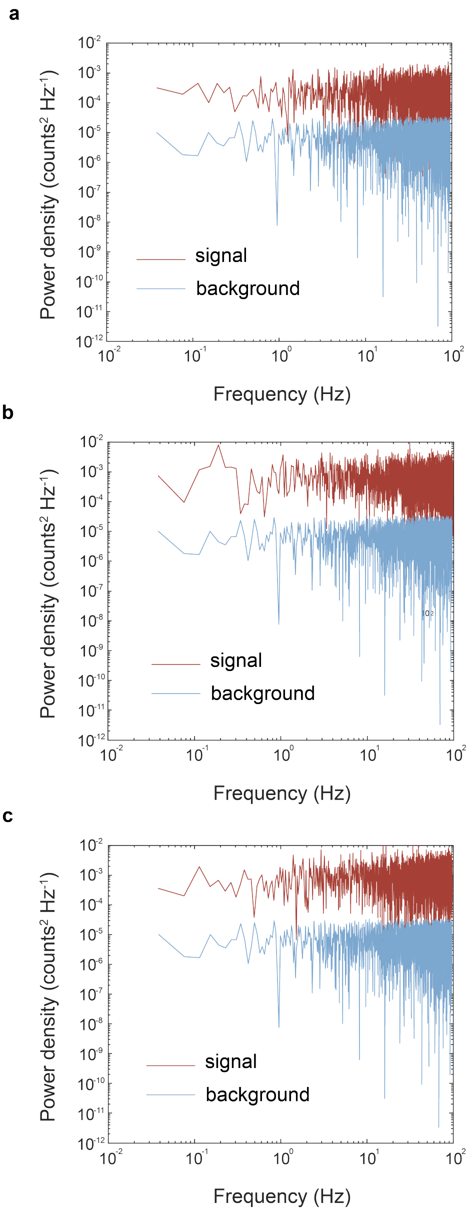

Suppl. Fig. 8. PSD analysis of single-molecule chemiluminescence signals at different luminol concentrations. (a-c) The concentration of luminol is 0.024 mM, 0.122 mM, 0.488 mM. Chemiluminescence reaction conditions: 2.25 mM H_2O_2, 0.23 mM 4-iodophenol, 2.5 nM HRP. Imaging condition: EM Gain: 500, exposure time: 0.005 s. Source data are provided as a Source Data file.

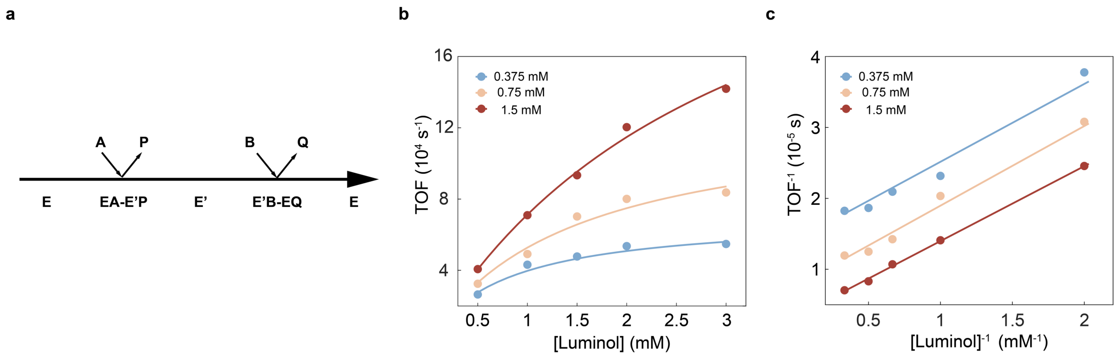

Suppl. Fig. 9. 'Ping-pong' double-substrate enzyme mechanism and the Lineweaver-Burk plot. (a) Double-substrate 'ping-pong' mechanism scheme. E: enzyme, E': enzyme intermediate, A: first substrate, B: second substrate, P: product from A, Q: product from B. **(b)** Michaelis-Menten equation fitting of 'ping-pong' mechanism at different luminol and H_2O_2 concentrations. **(c)** Single-molecule Lineweaver-Burk plot with TOF. HRP concentration: 125 nM. Source data are provided as a Source Data file.

Referring to Cleland, W.W. et al[1-2], the first substrate A combines with enzyme E to form EA, then the functional group of A is transferred to E to form E'P, which releases P to form E'. E' combines with the second substrate B following the same principles to form Q and release E[1]. In the whole reaction process, only the binary complex form exists, and the ternary complex form is absent. The 'ping-pong' mechanism follows,

$$\mathrm{TOF}^{-1} = \frac{1}{V} = \frac{K_\mathrm{m}^\mathrm{H}}{V_\mathrm{max}[\mathrm{H}]} + \frac{K_\mathrm{m}^\mathrm{L}}{V_\mathrm{max}[\mathrm{L}]} + \frac{1}{V_\mathrm{max}} \quad (1)$$

When the concentration of hydrogen peroxide is fixed, with the increase of luminol concentration, the TOF gradually decreases, and the reaction rate gradually increases and tends to saturation. Both K_m^L and V_max of luminol gradually increased with the increase of H_2O_2 concentration. The Lineweaver-Burk curves are linear and parallel to

each other at the three hydrogen peroxide concentrations, which are in line with the proposed ‘ping-pong’ mechanism.

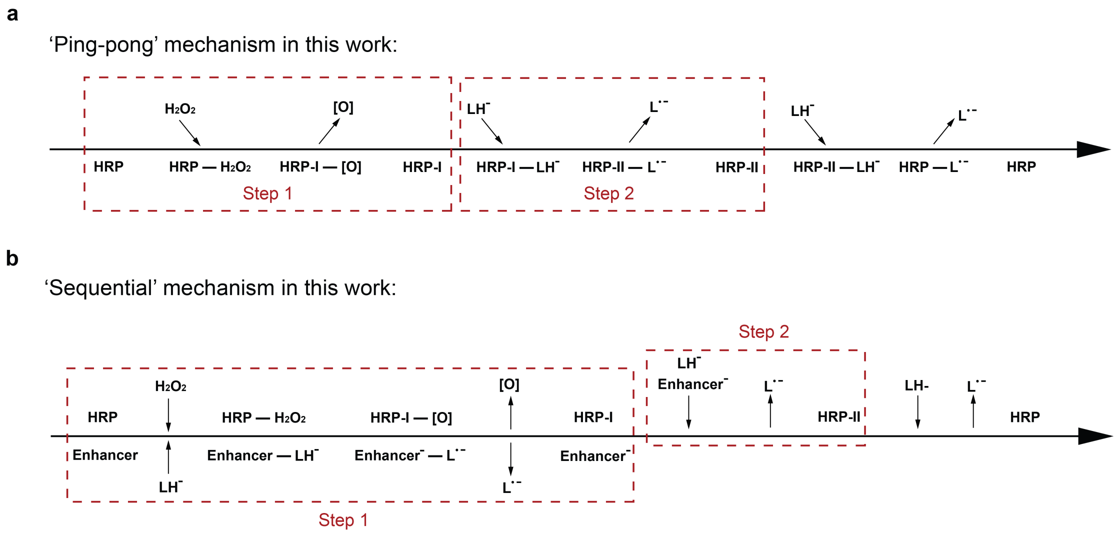

Suppl. Fig. 10. Reaction mechanism scheme in this work. (a) 'Ping-pong' reaction mechanism. **(b)** 'Sequential' reaction mechanism. The direction of the arrow is reflective of the direction of the reaction process. Step1: the first step of HRP-catalyzed reaction, Step2: the second step of HRP-catalyzed reaction, Enhancer: 4-iodophenol.

For the 'ping-pong' mechanism with the absence of 4-iodophenol, the substrate H_2O_2, binds to HRP to produce the [O] and HRP intermediates: HRP-I. And the subsequent substrate LH^-, then reacts with HRP-I and HRP-II to produce AP^{2-} and restore HRP to its initial form. However, in the 'sequential' mechanism (see Suppl. Fig. 6), substrates H_2O_2 and LH^- both have to bind to the HRP before releasing the products and restoring the HRP to its initial state, which is one reaction step less compared to the 'ping-pong' mechanism. In the first step, 4-iodophenol acts as a redox mediator to directly oxidize LH^- to $L^{\cdot-}$, resulting in the early oxidation of luminol[3], which advances and accelerates the processing of reaction and exhibits characteristics similar to the first step of the 'sequential' mechanism. Besides, 4-iodophenol's free radical intermediates then react with HRP-I to generate HRP-II and accelerate the enzyme turnover frequency so that more LH^- molecules are involved in the reaction

at the same time. In combination with the above two steps of acceleration and the early involvement of LH^- in the reaction, the kinetics of the reaction shows a mechanism shift.

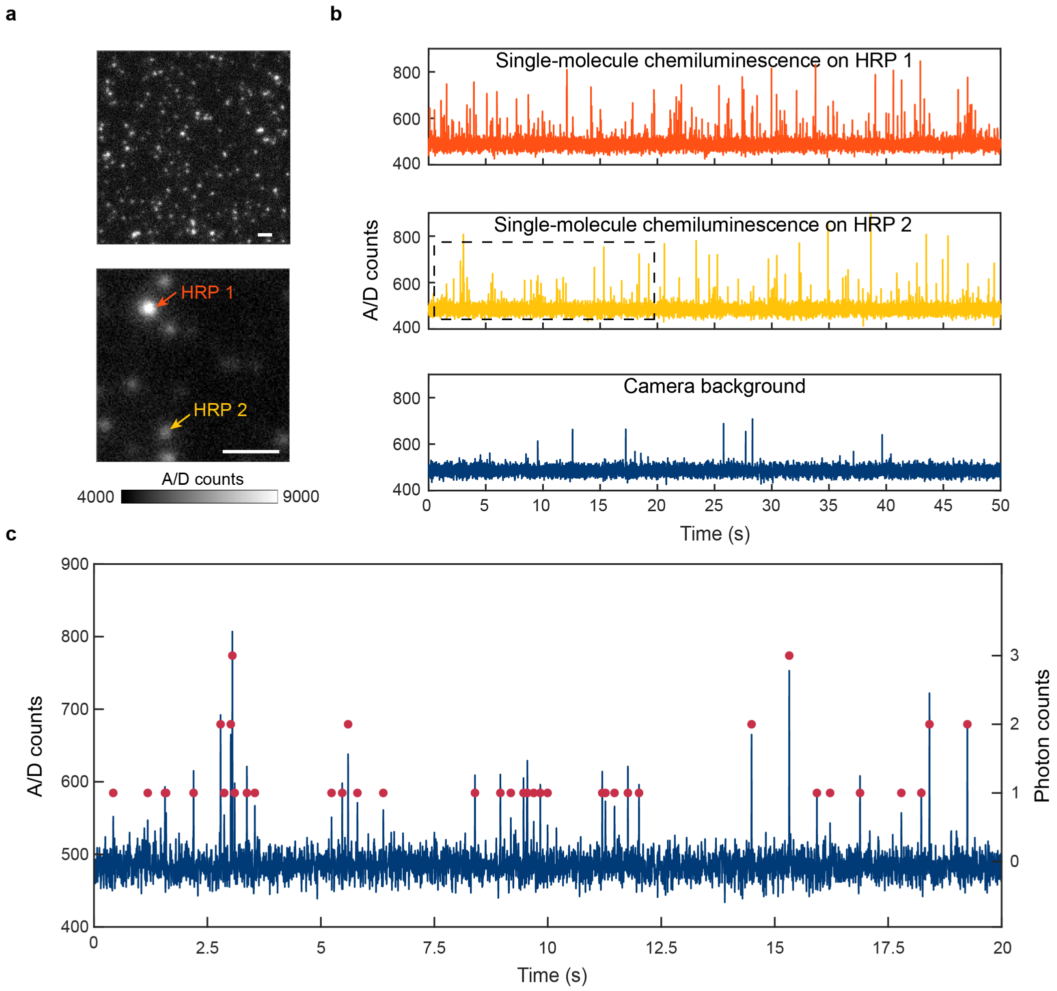

Suppl. Fig. 11. Catalytic behavior on single HRP particles. (a) Chemiluminescence of single HRP particles on glass coverslip. Exposure time: 10 s, EM Gain= 500, 60× oil objective. Scale bar: 10 μm. **(b)** Single molecule trajectories of two selected particles in **(a)** and camera background. Exposure time: 5 ms, EM Gain= 500. **(c)** Single-molecule chemiluminescence turnover trajectory of the selected area (black dashed box) in **(b)**. Source data are provided as a Source Data file.

Single HRP particles were deposited on the glass coverslip and two HRP particles were selected for characterization, as shown in Suppl. Fig. 11a. Suppl. Fig. 11b shows the single turnover signals of chemiluminescent reactions on single HRP particles. Compared with camera background, single-molecule chemiluminescence on catalyst

particles can be observed visually. In addition, we selected one portion of the trajectory (black dashed box) for further analysis (Suppl. Fig. 11c), and the results show that the catalytic behavior on a single HRP particle exhibits a fluctuation which is different from the bulk assay.

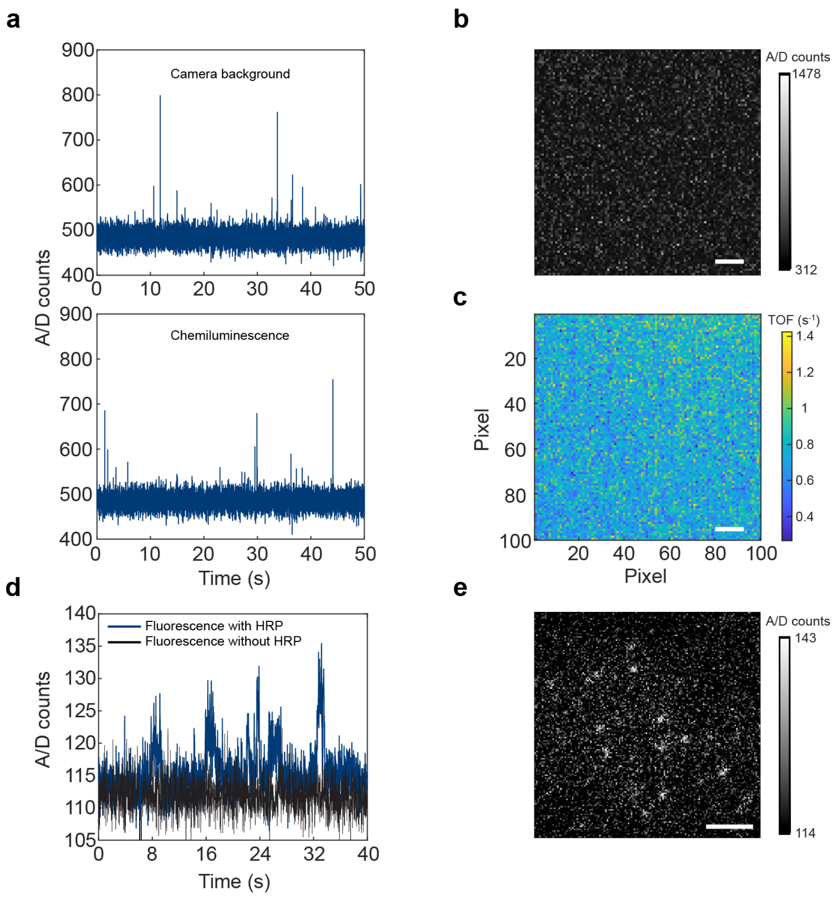

Suppl. Fig. 12. Immobilized HRP molecule detection in chemiluminescence and fluorescence on glass coverslip. (a) Single-molecule trajectory of camera background and chemiluminescence. Exposure time: 5 ms, EM Gain= 500. Luminol concentration: 200 μM, H_2O_2 concentration: 100 μM. **(b)** Chemiluminescence image of immobilized HRP on glass coverslip. Exposure time: 10 s, 100× oil objective, EM Gain= 500. Scale bar: 2 μm. **(c)** TOF analysis of selected 100×100 pixels in chemiluminescence of **(b).** Scale bar: 2 μm. **(d)** Intensity trajectory of fluorescence with a single HRP molecule and without HRP molecule. Exposure time: 20 ms. 100× oil objective. Amplex red concentration: 10 μM, H_2O_2 concentration: 100 nM. **(e)** Fluorescence image of single HRP molecules immobilized on glass coverslip. Scale bar: 2 μm. Source data are provided as a Source Data file.

In order to immobilize single HRP molecules on the glass coverslip, a series immobilization steps were implemented, which involves NaOH-ethanol solution, 10% TESPA and isobutyltrimethoxysilane (1:10,000 ratio) in DMSO solution, 10 nM DMS·2HCl and 1 nM HRP. In this way, single HRP molecules are fixed to the surface of the glass coverslip at a density of 0.2 molecule/μm^2. As shown in Suppl. Fig. 12a-c, no obvious chemiluminescence signals can be seen from only a single HRP molecule immobilized on the glass coverslip. In order to verify whether the HRP was indeed immobilized to the glass surface, amplex red, a fluorescence probe which can be catalyzed by HRP in the presence of H_2O_2 and emit fluorescence with an emission peak of 585 nm was used. Suppl. Fig. 12e shows the fluorescence signal with obvious scintillation, and the position of the single HRP molecule is faintly visible. The 'on-off' trace shown in Suppl. Fig. 12d is the typical single-molecule turnover trajectory, indicating that the single HRP molecule was successfully immobilized to the glass surface. However, single-molecule chemiluminescence imaging did not provide any obvious signal. We suppose that HRP is a catalyst for both fluorescence and chemiluminescence, and the luminescence efficiency of chemiluminescence is much lower (e.g. luminol's quantum yield, 1.1%-1.2%) than that of fluorescence system, resulting in the too weak chemiluminescence at the single-enzyme molecule level to be detected at present. In the future, if more high efficiency chemiluminescence substrates or additives can be developed, the single HRP molecule detection and kinetics analysis might be possible.

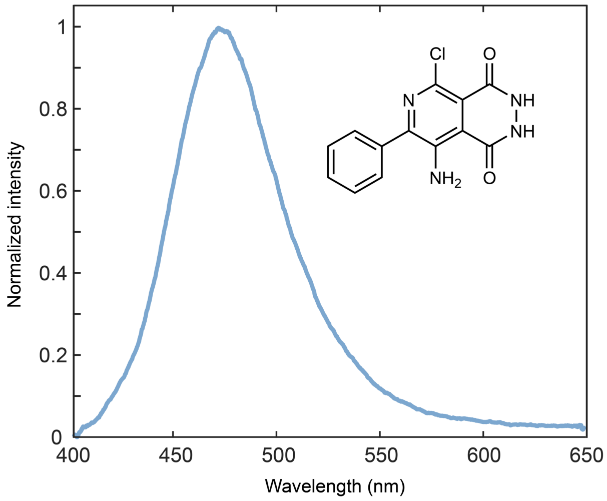

Suppl. Fig. 13. Chemiluminescence emission spectrum of L-012. The inset shows the molecular structure of L-012. Source data are provided as a Source Data file.

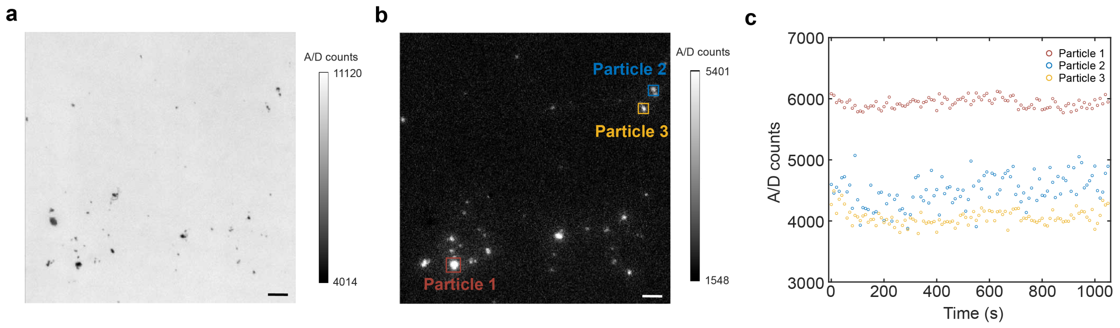

Suppl. Fig. 14. Stability of chemiluminescence intensity on hematin particles. (a) Bright field image of hematin particles. **(b)** Chemiluminescence image of hematin particles. **(c)** Chemiluminescence intensity stability of the selected particles. Scale bar: 10 μm. The chemiluminescence intensity on the particles remained stable over time, which laid the foundation for subsequent experiments. Source data are provided as a Source Data file.

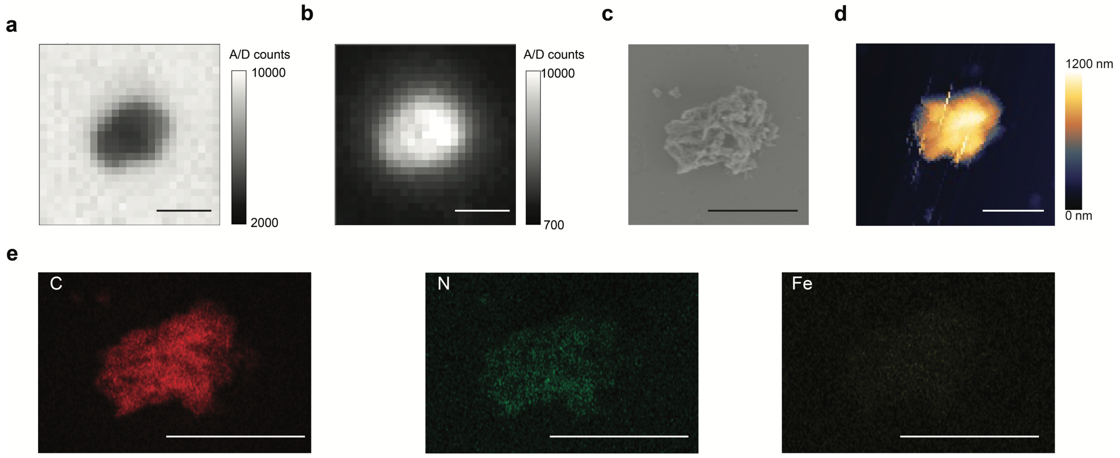

Suppl. Fig. 15. Correlated characterizations of a hematin particle. (a) Bright field, **(b)** chemiluminescence, **(c)** SEM, **(d)** AFM and **(e)** EDS characterizations. Scale bar: 2 μm. Chemiluminescence image is significantly diffused compared to bright field, SEM and AFM images. EDS mapping shows that the catalyst particles contain C, N, Fe, and the content of Fe is very low in comparison, which is in line with the element content distribution in the catalyst molecular formula.

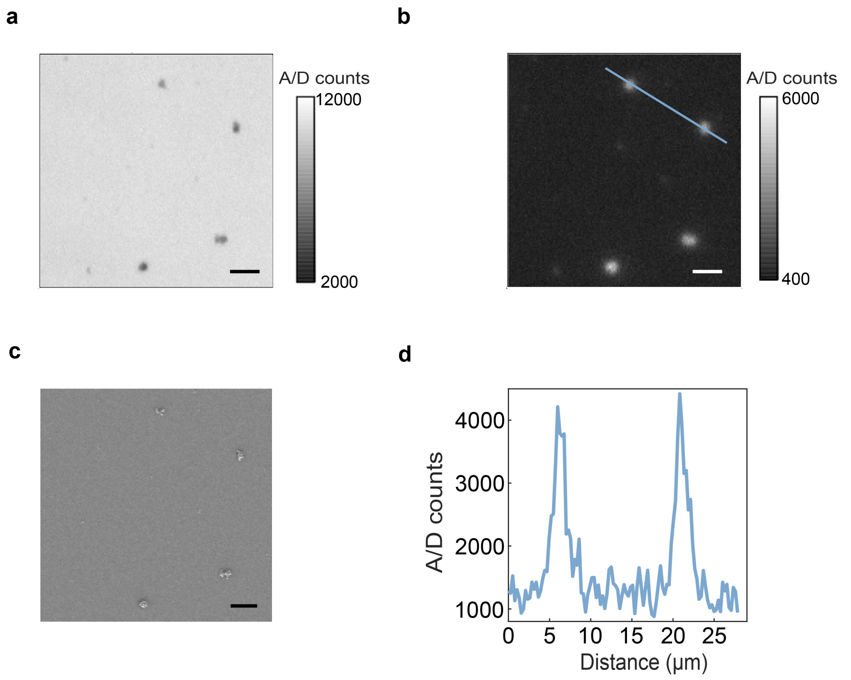

Suppl. Fig. 16. Correlated characterizations of four hematin particles. (a) Bright field, **(b)** chemiluminescence, **(c)** SEM images and **(d)** particles analysis along the blue line in (**b**). Scale bar: 5 μm. Source data are provided as a Source Data file.

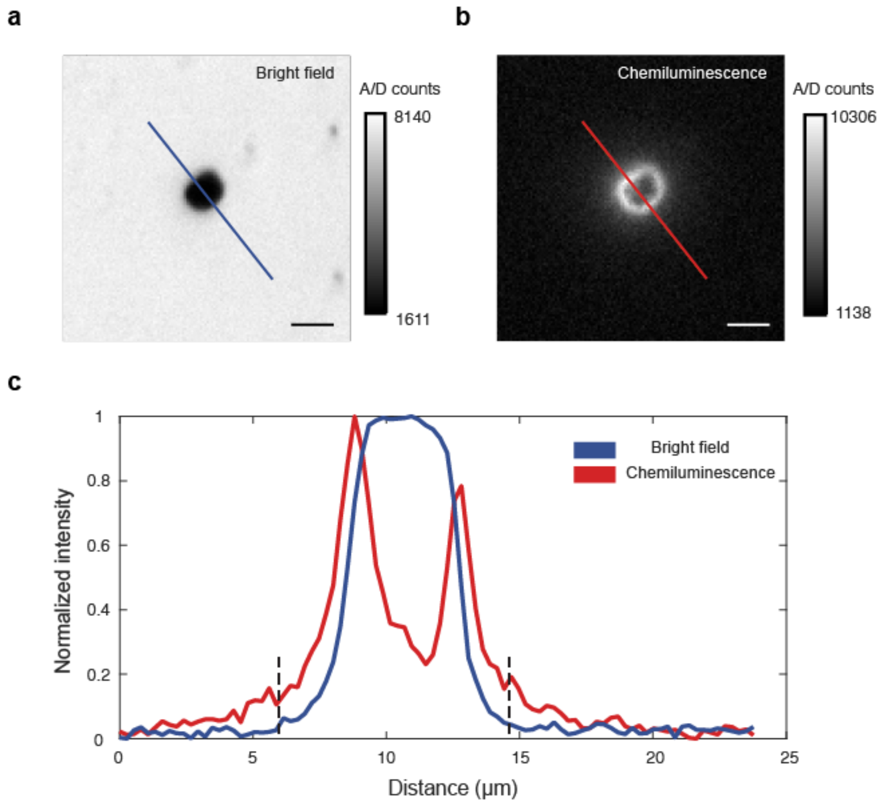

Suppl. Fig. 17. Chemiluminescence intensity distribution and diffusion analysis on the hematin particle. (a) Bright field and **(b)** chemiluminescence image of a hematin particle. Scale bar: 5 μm. **(c)** Normalized intensity profiles along the radial direction of **(a)** bright field and **(b)** chemiluminescence. Diffusion distance is from catalyst surface (the position of the black dashed line, and the normalized intensity of bright field is 0) to the position where the normalized chemiluminescence intensity is 0. Source data are provided as a Source Data file.

Obvious chemiluminescence diffusion was visible on the hematin catalysts. Suppl. Fig. 15a and b show the bright field and chemiluminescence image of the same hematin particle. Normalized intensity profiles along the radial direction are shown in Suppl. Fig. 15c. According to the distribution, chemiluminescence intensity decreases more slowly than that of bright field with the direction away from the catalyst surface. The

distance from the catalyst surface (the position that the normalized intensity of bright field is 0) to the position where the chemiluminescence intensity is 0 is considered to be the diffusion distance of radicals' intermediates. As shown in Suppl. Fig. 15c, the diffusion distance on the catalyst particles is about 5 μm, which is comparative to the previous result in the literature[4]. According to the equation:

$$L = \sqrt{2\mathrm{D}\tau} \quad (2)$$

where L is the diffusion distance, D is the diffusion coefficient of radicals, $\mathrm{D}_{\mathrm{L-012}} = 6.6\times10^{-6}\ \mathrm{cm}^2\ \mathrm{s}^{-1}$, $\mathrm{D}_{\mathrm{H_2O_2}} = 1\times10^{-5}\ \mathrm{cm}^2\ \mathrm{s}^{-1}$, and τ is the radical lifetime[4-6], the H_2O_2 intermediate's radical lifetime is estimated to be 12.5 ms and L-012 intermediate's radical lifetime is 18.9 ms.

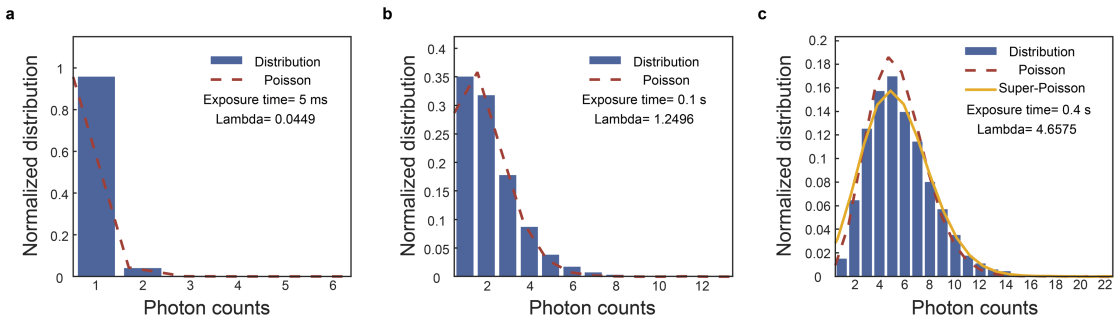

Suppl. Fig. 18. Photon count distribution on a single hematin particle. (a-c) Distribution of photon number at exposure time of 0.005 s, 0.1 s and 0.4 s. As the exposure time increases, the number of photons detected on the catalyst particles increases gradually and follows the Poisson distribution model well. The lambda is 0.0449, 1.2496 and 4.6575. Source data are provided as a Source Data file.

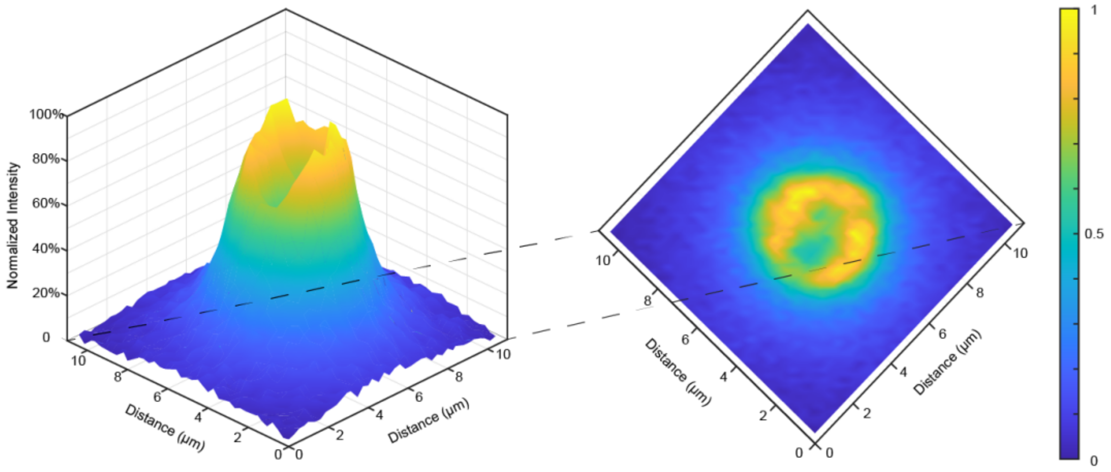

Suppl. Fig. 19. Chemiluminescence intensity distribution for the single particle in Fig. 3e. Source data are provided as a Source Data file.

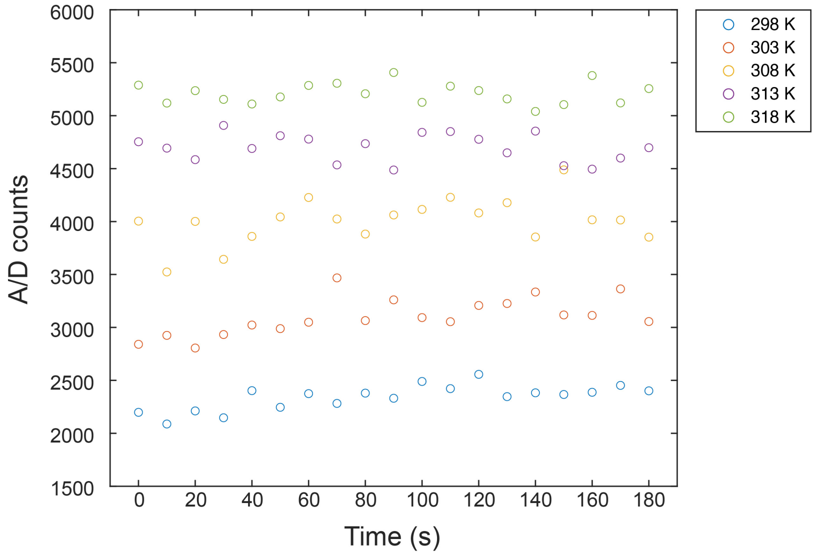

Suppl. Fig. 20. Chemiluminescence stability on the hematin particle. Exposure time: 10 s, EM Gain: 500. Source data are provided as a Source Data file.

Suppl. Table 1. Abbreviations and corresponding explanations.

Abbreviations	Name	Structural formula
LH^-	luminol (5-amino-2,3-dihydro-1,4-phthalazinedione)	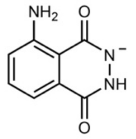
$L^{\cdot-}$	luminol ionic radical	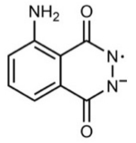
AP^{2-}	3-aminophthalic acid	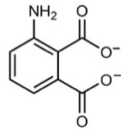
HRP	horseradish peroxidase	PDB DOI for HRP: https://doi.org/10.2210/pdb1H58/pdb
HRP-I, HRP-II	peroxidase reactive intermediates	PDB DOI for HRP-I: https://doi.org/10.2210/pdb1HCH/pdb PDB DOI for HRP-II: https://doi.org/10.2210/pdb1H55/pdb
[O]	the reactive oxygen species for H_2O_2 decomposition	$HO_2^-/O_2^{\cdot-}$
L-012	8-amino-5-chloro-7-phenylpyrido[3,4-d]pyridazine-1,4(2H,3H)dione	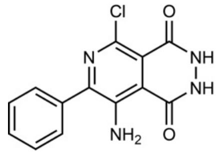
Product of oxidized L-012	8-amino-5-chloro-7-phenylpyrido[3,4-d]pyridazine-1,4(2H,3H)dicarboxylic acid	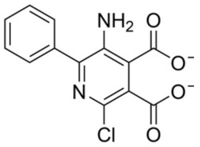

Suppl. Table 2. Calculated parameters of 'sequential' mechanism in Fig. 2f.

H_2O_2 concentration (mM)	K_m^L (mM)	K_{iH}	V_{max} (s^{-1})	k_{cat} (mL μg^{-1} s^{-1})
0.15	0.05915	0.03153	2.466	25.05
0.75	0.06221	0.05229	8.768	89.02
2.25	0.0838	0.07324	17.6	178.68

$$k_{cat} = \frac{V_{max}}{[E]} \quad (3)$$

[E] is the concentration of HRP, and k_{cat} represents the catalytic constant.

Suppl. Table 3. Calculated parameters of 'ping-pong' mechanism in Suppl. Fig. 9.

H_2O_2 concentration (mM)	K_m^L(mM)	V_{max}(s^{-1})	k_{cat}(mL μg^{-1} s^{-1})
0.3	0.8132	3.962	0.7924
0.6	1.511	7.261	1.4522
1.2	2.736	14.87	2.974

Supplementary References

1. Cook, P.F. & Cleland, W.W. *Enzyme Kinetics and Mechanism* (Garland Science, 2007).

2. Cleland, W.W. The kinetics of enzyme-catalyzed reactions with two or more substrates or products: III. Prediction of initial velocity and inhibition patterns by inspection. *Biochim. Biophys. Acta(BBA)-Spec. Sect. Enzymol. Subj.* **67**, 188-196 (1963).

3. Easton, P.M. et al. Quantitative model of the enhancement of peroxidase-induced luminol luminescence. *J. Am. Chem. Soc.* **118**, 6619-6624 (1996).

4. Zhou, P., Hu, S., Guo, W. & Su, B. Deciphering electrochemiluminescence generation from luminol and hydrogen peroxide by imaging light emitting layer. *Fundam. Res.* **2**, 682-687 (2022).

5. van Stroe-Blezen, S. A. M., Everaerts, F. M., Janssen, L. J. J. & Tacken, R. A. Diffusion coefficients of oxygen, hydrogen peroxide and glucose in a hydrogel. *Anal. Chim. Acta*, **273**, 553-560 (1993).

6. Koizumi, Y. & Nosaka, Y. Kinetics simulation of luminol chemiluminescence based on quantitative analysis of photons generated in electrochemical oxidation. *J Phys Chem A.* **117**, 7705-7711 (2013).
