# Supplementary figures and images for: Direct probing of single-molecule chemiluminescent reaction dynamics under catalytic conditions in solution
Source: Nat Commun. 2023 Dec 2;14:7993. doi: 10.1038/s41467-023-43640-1 (PMC10693624; doi:10.1038/s41467-023-43640-1)

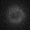

Supplement: Supplementary file 4 — Source Data [file 41467_2023_43640_MOESM4_ESM.zip › Source Data/Suppl.Fig.19/Suppl.Fig.19-direct chemiluminescence mapping.tif]
